# Mechanical circulatory support for cardiovascular complications in a young COVID‐19 patient

**DOI:** 10.1111/jocs.14916

**Published:** 2020-08-02

**Authors:** Aron Frederik Popov, R. Berger, Christian Schlensak, Malte N. Bongers, Helene Haeberle, Metesh Acharya, Henning F. Lausberg

**Affiliations:** ^1^ Department of Cardiothoracic and Vascular Surgery University of Tuebingen, Eberhard‐Karls‐University Tübingen Tübingen Germany; ^2^ Department of Diagnostic and Interventional Radiology University of Tuebingen, Eberhard‐Karls‐University Tübingen Tübingen Germany; ^3^ Department of Anaesthesiology and Intensive Care Medicine University of Tübingen, Eberhard‐Karls‐University Tübingen Tübingen Germany; ^4^ Department of Cardiac Surgery Glenfield Hospital Leicester UK

**Keywords:** ARDS, COVID‐19, ECMO, ischemic stroke, right heart failure

## Abstract

**Background:** The current coronavirus (COVID‐19) pandemic is associated with severe pulmonary and cardiovascular complications.

**Case presentation:** This report describes a young patient with COVID‐19 without any comorbidity presenting with severe cardiovascular complications, manifesting with pulmonary embolism, embolic stroke, and right heart failure.

**Conclusion:** Management with short‐term mechanical circulatory support, including different cannulation strategies, resulted in a successful outcome despite his critical cardiovascular status.

## 1 INTRODUCTION

The novel coronavirus (COVID‐19) has been identified as the cause of a severe acute respiratory syndrome, accounting for thousands of cases of severe pneumonia, respiratory failure and death globally.^1^ Although current management is largely supportive, a minority with cardio‐circulatory instability secondary to acute cardiac injury, myocarditis, acute pulmonary embolism, or other complex conditions,^2^ require mechanical ventilation or extracorporeal membrane oxygenation.

## 2 CASE REPORT

**Figure 1:**
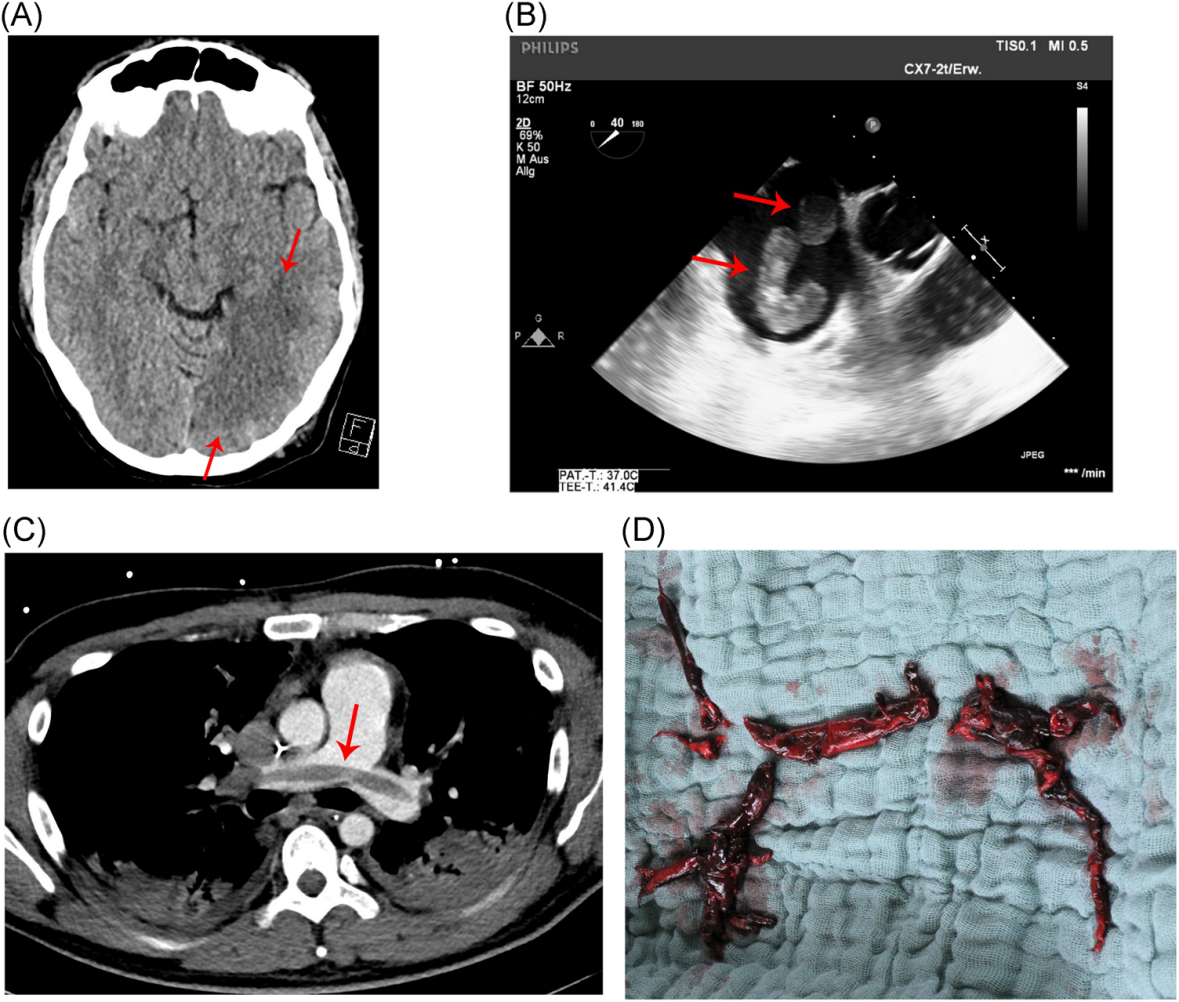
A, Contrast‐enhanced computed tomography (CT) scan of brain demonstrating left‐sided ischemic stroke. B, CT scan of chest demonstrating a large pulmonary embolus extending into the main pulmonary arteries. C, Trans‐esophageal echocardiogram demonstrating thrombus formation (red arrows) within the right ventricle. D, Multiple thrombi retrieved from pulmonary artery intra‐operatively

A 33‐year old man without any known comorbidities presented with dyspnoea to our tertiary center. He tested positive for COVID‐19 2 weeks before admission during a business trip in Asia. With progressive deterioration in his lung function, he was intubated and mechanically ventilated in a prone position in accordance with guidelines for the management of Acute Respiratory Distress Syndrome (ARDS). Brain and thoracic computed tomography (CT) scanning were performed to investigate a persistent neurological deficit following sedation withdrawal, and demonstrated a large left‐sided ischemic stroke (Figure 1A) with extensive central pulmonary embolism (Figure 1B). The patient subsequently developed a sudden reduction in cardiac output, when emergency trans‐esophageal echocardiography revealed right heart failure with thrombus formation in the right atrium and right ventricle (Figure 1C, Videos 1 and 2). A patent foramen ovale was excluded.

The patient was urgently transferred to the operating theatre for pulmonary embolectomy and additionally to establish salvage veno‐arterial extracorporeal membrane oxygenation (VA‐ECMO) for fulminant right heart failure. At surgery, multiple formations of pulmonary thrombi were retrieved (Figure 1D). The following day, re‐thoracotomy was necessary for evacuation of a mediastinal hematoma, as well as to upgrade from VA‐ECMO to veno‐arterial‐venous ECMO due to ongoing respiratory failure despite VA‐ECMO therapy. The patient also developed arterial embolism affecting his left hand, which was treated conservatively in view of adequate anticoagulation with unfractionated heparin while receiving mechanical circulatory support (MCS), and on account of his complex hemodynamic instability. On the second postoperative day, continuous veno‐venous hemodialysis was commenced for acute kidney injury.

The arterial ECMO cannula was removed 1 week after the initial surgery, following recovery of right heart function, and veno‐venous ECMO (VV‐ECMO) was maintained for another 10 days. An improvement of lung function and neurological status during VV‐ECMO and prone ventilator therapy were observed. The patient was subsequently extubated, and 3 weeks after initial surgery, he was discharged to a rehabilitation unit with right‐sided hemiparesis and leg weakness. During his intensive care unit stay, a comprehensive haematology screen did not yield evidence of any underlying hypercoagulable disorder. According to the standard in our centre, the patient was anticoagulated with continuous intravenous heparin with a goal partial thromboplastin time level of 50 to 70 seconds through the ECMO therapy.

## 3 COMMENT

The use of ECMO has been advocated to sustain respiratory and/or cardiovascular function and might represent the only effective intervention in the difficult circumstances of circulatory instability in COVID‐positive cases. The COVID‐BioB Study Group reported that older age, cardiovascular disease, chronic lung disease, hypertension, diabetes, and obesity are associated with worse outcomes.^3^ These risk factors, however, were not present in our young patient who nevertheless developed serious cardiovascular complications with pulmonary embolism, embolic stroke, and right heart failure. Indeed, some patients with COVID‐19 infection will have a high incidence of venous and arterial thromboembolism within an intensive care setting, which may lead to fatal cardio‐circulatory events.^4^ Interestingly, it was recently reported that COVID‐19 infection is associated with large‐vessel stroke in patients younger than 50 years^5^ as observed in our patient, which may be attributable to coagulopathy and vascular endothelial dysfunction.^6^

ECMO is traditionally utilized as rescue therapy in the most severe cases of refractory cardiorespiratory failure. However, it is associated with significant neurological, vascular, renal and hematological adverse effects, including intracerebral hemorrhage, stroke, limb ischemia and procoagulant states. As evidenced in this report, we successfully employed various ECMO strategies, even in the challenging context of contemporaneous acute ischemic stroke, which could risk hemorrhagic conversion, central pulmonary embolism, kidney injury and upper limb ischemia, following high‐risk pulmonary endarterectomy in an unstable patient. We've followed the standards of anticoagulation for circulatory support patients.^7^ Thus, the judicious use of ECMO in carefully selected patient cohorts in experienced centers may be of great benefit to, and achieve favorable clinical outcomes in patients developing cardiorespiratory complications during the current COVID‐19 era. ECMO should be perceived as an accessible and highly valuable tool in the clinician's armamentarium, rather than a “last resort” option in apparently futile cases.

In conclusion, we report a successful outcome in a young patient who underwent short‐term MCS and high‐risk cardiothoracic surgery for the treatment of acute right heart failure with severe pulmonary embolism and large‐vessel embolic stroke as a complication of COVID‐19 infection. Short‐term MCS with different cannulation strategies may represent a viable treatment modality for cardiovascular complications with venous and arterial thromboembolism in patients with COVID‐19 infection.

## CONFLICT OF INTERESTS

The authors declare that there are no conflict of interests.

## ETHICS STATEMENT

Informed consent was obtained from the patient. Local institutional ethical processes were followed.

## Supporting information

Supporting information

Supporting information
